# Impact of Elderly Acute Care Discharge Services on Prevention of Rehospitalisation: A Retrospective Cohort Study Using National Health Data from Kita Ward, Tokyo

**DOI:** 10.5334/ijic.8913

**Published:** 2025-02-04

**Authors:** Masumi Takei, Satoshi Miyata, Mariko Inoue, Kenzo Takahashi

**Affiliations:** 1 Faculty of Nursing, Shonan Kamakura University of Medical Sciences, Kanagawa, Japan; 2 Graduate School of Public Health, Teikyo University, Tokyo, Japan; 3 Tetsuikai Research Institute, Tokyo, Japan; 4 Department of Pediatrics, Navitas Clinic Kawasaki, Kanagawa, Japan

**Keywords:** community-based integrated care, hospital discharge services, readmission, elderly acute care, survival analysis

## Abstract

**Introduction:**

Integrated care poses a significant challenge for healthcare policies in Japan as evaluation of hospital discharge services is limited. This study aimed to elucidate the effects of discharge services for elderly acute-care patients on preventing rehospitalisation.

**Methods:**

A retrospective cohort study was conducted using national health data from Kita Ward, Tokyo. Survival analysis was performed with a Cox proportional hazards model, with readmission hazard ratios (HRs) as the primary endpoint. Subgroup analysis examined interactions between each discharge service category (dummy variable) and readmission.

**Results:**

The study encompassed 6,681 subjects. The Cox model adjusted for age, gender, and complications revealed increased readmission events in the discharge service group (HR = 2.92, 95% CI 2.60–3.27). Subgroup analysis by age and length of hospital stay identified a preventive effect in the 85-year-old group (HR = 0.68, 95% CI 0.49–0.93) and 15–21-day length of stay group (HR = 0.73, 95% CI 0.53–1.01), suggesting that discharge services may inadvertently lower barriers to readmission due to healthcare system influences.

**Conclusion:**

While discharge services may elevate readmission demand, they appear to have a preventive effect for individuals aged 85 and over or with an average length of stay of 15–21 days.

## 1 Introduction

Japan’s population aged 65 and over has reached 28.8%, and by 2065, one of every 2.6 persons will be aged 65 and over [1]. This rapid aging of the population is attributed to the proliferation of the universal health insurance system and the implementation of interventions by the elderly health and welfare system [2]. Population aging correlates with a heightened demand for medical and long-term care services [3,4,5]. Readmission to hospitals in the US has garnered attention from policymakers as an indicator of enhanced quality of care and is associated with a significant financial burden [6]. The Japanese government has been actively promoting a division of roles and cooperation of medical functions and shorter hospital stays through the enhancement of discharge services. Additionally, it advocates for a transition from hospital-centric medical care to a community-based integrated care system encompassing home care and informal resident networks, particularly focusing on coordination of medical care for the elderly [7,8,9,10].

To facilitate the provision of discharge services, Japanese healthcare policies have been updated to adjust incentives for payment of medical services by health insurance that correspond to the revenue of the medical institutions. This strategic move aims to encourage the allocation of personnel to discharge service departments [11,12]. Moreover, in comparison to hospital-based interventions, strategies associated with community-based care have suggested efficacy in mitigating emergency department utilisation among the elderly population [13]. Discharged elderly patients are relocated to their homes, long-term care insurance facilities, or other residential settings for the elderly, with the nature of their discharge needs varying according to the functional capacity of the ward [14]. The outcomes of discharge services are multifaceted, including reduced length of stay, decreased rates of unplanned readmissions, heightened satisfaction levels among patients, families, and healthcare professionals, and optimised utilisation and cost-effectiveness of social resources [15]. Efforts to diminish readmission rates have underscored the significance of discharge communication interventions [16], effects of transitional care including home visits [17,18,19], and social characteristics and support mechanisms [20,21]. In Japan, although no significant association has been reported between discharge services, such as planning, coordination, and rehabilitation prescription at discharge, and 30-day readmission rates among the latter-stage for elderly [22], a notable correlation has been observed with reduced readmission rates among the elderly with dementia and fractures in long-term care facilities [23].

The Japanese healthcare system is characterised by a universal health insurance system, social insurance system, and free access, potentially reducing the barrier for hospitalisation compared to other nations. Evaluating the nuances of implementing discharge services in acute care settings and identifying effective strategies for reducing readmissions hold significance for advancing a comprehensive community care framework. Yet, to date, there exists a paucity of multicentre, longitudinal studies utilising municipal medical data in Japan.

In this study, we conducted an analysis of national health data of elderly patients in Kita Ward, Tokyo, utilising survival time analysis. Our specific objectives were 1) to scrutinise the characteristics of elderly patients with and without discharge services in acute care wards; 2) to investigate the hazard ratio [HR] of readmission events pertaining to the entirety of discharge services as well as individual discharge services, with the group not receiving discharge services serving as the control; and 3) to explore the impact of discharge services in terms of gender, age, and length of stay through subgroup analyses.

## 2 Methods

### 2.1 Study site

This study was conducted in Kita Ward, Tokyo, Japan. Among the 23 wards of Tokyo, Kita Ward has the highest percentage of people aged 65 and over, with 24.7% in 2021, whereas the average for the whole of Tokyo prefecture is 22.7%, and that for Japan is 28.6% [24]. In the medical service coverage area that includes Kita Ward, 68.5% of patients receive medical care in a hospital with acute care facilities, and when the adjacent areas of Tokyo are included, that rate rises to 91.9%, the highest rate in Tokyo. As two university hospitals in the suburbs of Kita Ward provide advanced medical care, there is easy access to hospitals with acute medical care [25]. This study contributes to an improvement project on discharge support in Kita Ward of Tokyo, where the elderly highest population has a high demand for hospitalisation and access to abundant acute wards.

### 2.2 Study population

The study cohort comprised elderly men and women aged 65 years or above residing in Kita Ward, Tokyo. Data were sourced from a large, anonymised receipt database obtained from Kita Ward, Tokyo, which oversees the National Health Insurance scheme for individuals aged 65 years and older. Therefore, the dataset encompasses information from all inhabitants of Kita Ward who are enrolled in the National Health Insurance programme and are aged 65 years and older, as well as those aged 75 years and above. Included in the dataset are patient-level sociodemographic characteristics, details of medical facilities utilised, prescribed medications, and diagnoses recorded during clinical visits for insurance claims purposes. Diagnoses were coded in accordance with the International Classification of Diseases, Tenth Revision (ICD-10).

**Figure 1:**
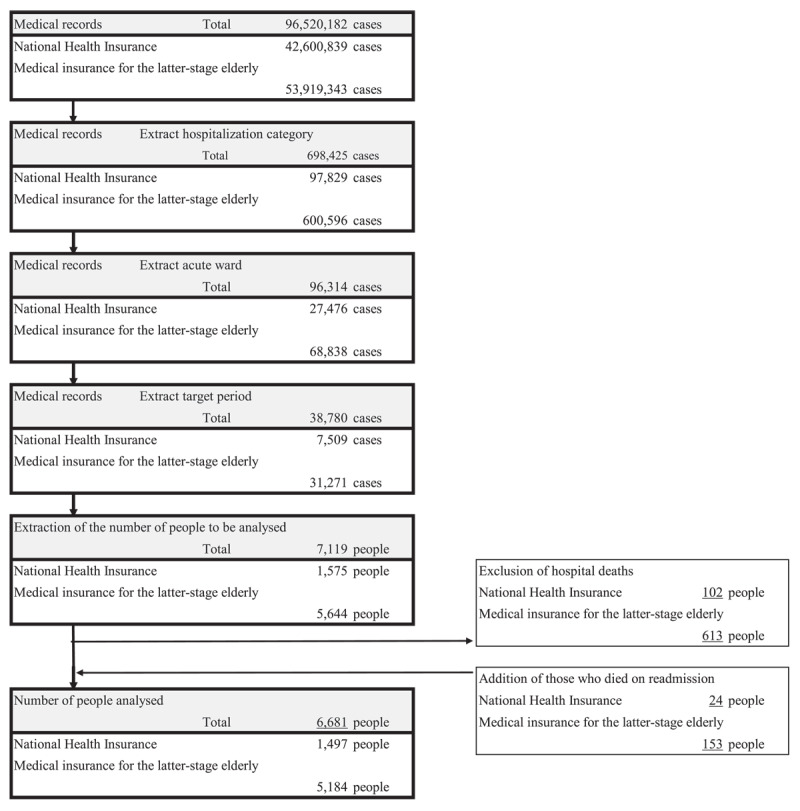
Flow of participant extraction process.

Patients aged 65 years and above who underwent outpatient, inpatient, or home health care from a medical facility between 1 April 2016 and 31 March 2018 and who received discharge services in acute care wards were included in the study. Acute care wards were delineated as wards with a nurse-to-patient staffing ratio of either one nurse for every seven or ten patients. Selection of patients was carried out based on their hospitalisation status, placement in acute care wards, and the timeframe of the study period, whereas individuals who died during their initial admission within the covered period were excluded. Following the exclusion of subjects who died subsequent to readmission, the final cohort analysed consisted of 6,681 patients. The process of selecting the subjects for analysis is described in Figure 1.

### 2.3 Data analysis

For the initial objective, a descriptive statistical analysis was used to compare patient characteristics between those receiving and not receiving discharge services. Differences in these characteristics were evaluated using Fisher’s exact test for categorical variables, including gender, age, length of hospital stay, utilisation of home health care prior to admission, pre-admission facility usage, admission due to injury or illness, readmission rates, and end-of-life care at home. The expected age values were compared using Welch’s *t*-test, whereas comparisons of the lengths of hospital stay and observation were conducted using the Wilcoxon rank sum test, incorporating Yates’ continuous correction, with a significance threshold set at 0.05.

For the second objective, the log-rank test was used in univariable analysis to compare the event rate between patients with and without discharge services. Furthermore, a Cox proportional hazards model was used to compute the multivariable-adjusted HR and its corresponding 95% confidence interval [CI], incorporating age, gender, complications, and length of hospital stay as covariates. The primary endpoint of this retrospective cohort study was to determine the HR of readmission events associated with discharge services and its subcomponents throughout the observation period, commencing from the date of discharge, with the no discharge service group serving as the reference (HR = 1). In terms of complications, the model fit was assessed, and binary data categorised as 4 or fewer and 5 or more complications were used. The subcomponents of discharge services were classified into three categories, as referenced in a prior study [21]: discharge planning, coordination with community care, and rehabilitation discharge instruction.

For the third objective, we explored the effects of discharge services across subgroups defined by gender, age, and length of hospital stay as a secondary endpoint. To ascertain that the impacts of discharge services varied across the study cohort, we evaluated the significance of the interaction between age, gender, and length of stay categories (represented as dummy variables) and discharge services (coded as group = 1 for with services and group = 0 for without services). The significance level of interaction was established at p < 0.1. Independent effects within subgroups were examined through a combination analysis of the discharge service subcomponents. The combinations analysed included intervention only (encompassing discharge services and its subcomponents), intervention and age, intervention and gender, and intervention and length of hospital stay.

The endpoint of the observation period was delineated as the date of readmission, with censored data encompassing cases with no recorded outcomes, instances of end-of-life care at home, and deaths during hospitalisation at the point of readmission throughout the study duration. The evaluation of the project to improve discharge support through the involvement of people with lived experience is suggested by the results on readmissions based on the project design based on medical and long-term care integration.

Statistical analysis was performed using R language Ver. 4.3.0. R Core Team (2022). R: A language and environment for statistical computing (R Foundation for Statistical Computing, Vienna, Austria. https://www.R-project.org/).

### 2.4 Independent variables of interest: discharge services

We delineated three categories of discharge services encompassed by the Japanese medical insurance system during the study period, drawing upon insights from prior research: 1) discharge planning; 2) coordination with community care; and 3) rehabilitation discharge instruction.

Discharge planning aims to mitigate obstacles to discharge, whether to home, care facilities, or alternative hospitals. Within this framework, nurses and medical social workers within discharge planning units identify patients facing potential discharge impediments due to medical, physical, or environmental issues. They then formulate individualised discharge plans tailored to each patient’s needs, offering the requisite support to ensure a secure discharge [12]. For discharge planning, the target patient requirements include the specific illnesses of malignancy, dementia, and acute respiratory disease.

The objective of coordination with community care is to streamline collaboration between hospital personnel and community care managers by crafting long-term care strategies for patients based on their medical, physical, or environmental circumstances. Such coordination entails facility prerequisites concerning engagement with medical and nursing care establishments and businesses within the community’s integrated care system. By adhering to these prerequisites, medical institutions become eligible for reimbursement associated with coordination with community care [22].

Rehabilitation discharge instruction seeks to empower patients and their families through education, fostering self-management skills, averting functional decline, and enhancing independence in daily activities. This service is provided after discharge by physical therapists, occupational therapists, and speech-language pathologists in conjunction with physicians, nurses, and medical social workers [22].

### 2.5 Ethical consideration

This study was approved by the Teikyo University School of Medicine Ethics Review Committee (No. 20-144-2).

## 3 Results

### 3.1 Characteristics of the subjects

Of the total cohort, 3,906 patients (58.5%) received discharge services, a proportion significantly higher (p < 0.001) when considering the number of pre-existing conditions, length of hospital stay, the prevalence of patients receiving home care prior to admission, and those institutionalised before admission. The mean age ± standard deviation (SD) for the entire sample was 81.3 ± 7.8 years, with 2,900 patients (43.4%) being male. No significant differences were observed based on discharge service status. The numbers of patients using discharge services were categorised as follows: 1,263 patients (18.9%) for discharge planning, 3,378 (50.6%) for coordination with community care, and 1,374 (20.6%) for rehabilitation discharge instruction.

The total number of patients readmitted to the hospital stood at 1,872 (28.0%), a figure significantly higher in the discharge service group (p < 0.001). Furthermore, 199 patients (3.0%) died at home under the care of a home doctor, with this number also being notably higher in the discharge service group (p < 0.001). Conversely, no significant difference was observed in in-hospital mortality upon readmission (p = 0.301). A comparative analysis of patient characteristics for those with and without discharge services is presented in Table 1.

**Table 1:** Patient characteristics and outcomes with and without discharge services.*


	TOTAL (n = 6,681)				WITHOUT DISCHARGE SERVICES (n = 2,775)				WITH DISCHARGE SERVICES: (n = 3,906)				p-VALUE
		
	NO.	(%)	MEAN (SD)	MEDIAN (IQR)	NO.	(%)	MEAN (SD)	MEDIAN (IQR)	NO.	(%)	MEAN (SD)	MEDIAN (IQR)	

Age, years			81.3 (7.83)				81.1 (7.82)				81.4 (7.84)		0.13

65–74	1,535	(23.0)			682	(24.6)			853	(21.9)			0.03

75–84	2,874	(43.1)			1,183	(42.7)			1,691	(43.3)			

≥85	2,265	(33.9)			908	(32.7)			1,357	(34.8)			

Sex													

Male	2,900	(43.4)			1,195	(43.1)			1,705	(43.7)			0.63

Female	3,781	(56.6)			1,580	(56.9)			2,201	(56.3)			

Number of complications													

>5	3,378	(50.6)			1,185	(42.7)			2,185	(55.9)			<0.001

Length of stay, days			11.4 (8.61)	9 (4, 17)			10.5 (8.74)	8(3, 16)			12.1 (8.46)	10 (5, 18)	<0.001

<7	2,519	(37.7)			1,229	(44.3)			1,290	(33.0)			<0.001

7–14	2,026	(30.3)			755	(27.2)			1,271	(32.5)			

15–21	1,045	(15.6)			368	(13.3)			677	(17.3)			

≥22	1,091	(16.3)			423	(15.2)			668	(17.1)			

Pre-hospital home care users	806	(12.1)			257	(9.3)			549	(14.1)			<0.001

Pre-hospital facility users	665	(10.0)			198	(7.1)			467	(12.0)			<0.001

Discharge services													

Discharge planning	1,263	(18.9)							1,263	(32.3)			<0.001

Coordination with community care	3,378	(50.6)							3,378	(86.5)			<0.001

Rehabilitation discharge instruction	1,374	(20.6)							1,374	(35.2)			<0.001

Readmission	1,872	(28.0)			377	(13.6)			1,495	(38.3)			<0.001

Confirmation of death by home doctor after discharge	199	(3.0)			37	(1.3)			162	(4.1)			<0.001

In-hospital death on readmission	38	(0.7)			18	(0.8)			20	(0.6)			0.301


SD = standard deviation; IQR = interquartile range. * Indicates Fisher’s exact test, Welch’s t test for comparison of expected values for ages, and Wilcoxon rank sum test for length of stay. § Includes the absence of data for 3 months after the end of the person-days censoring.

### 3.2 Primary outcomes

Using the occurrence of readmission events in the group without discharge services as a reference (HR = 1), the rate of readmission events in the group receiving discharge services was elevated (HR = 2.92, 95% CI 2.60–3.27). When examining individual discharge services, the HRs were as follows: discharge planning HR = 2.02 (95% CI 1.83–2.23), coordination with community care HR = 2.83 (95% CI 2.55–3.14), and rehabilitation discharge instruction HR = 1.77 (95% CI 1.60–1.95).

**Figure 2:**
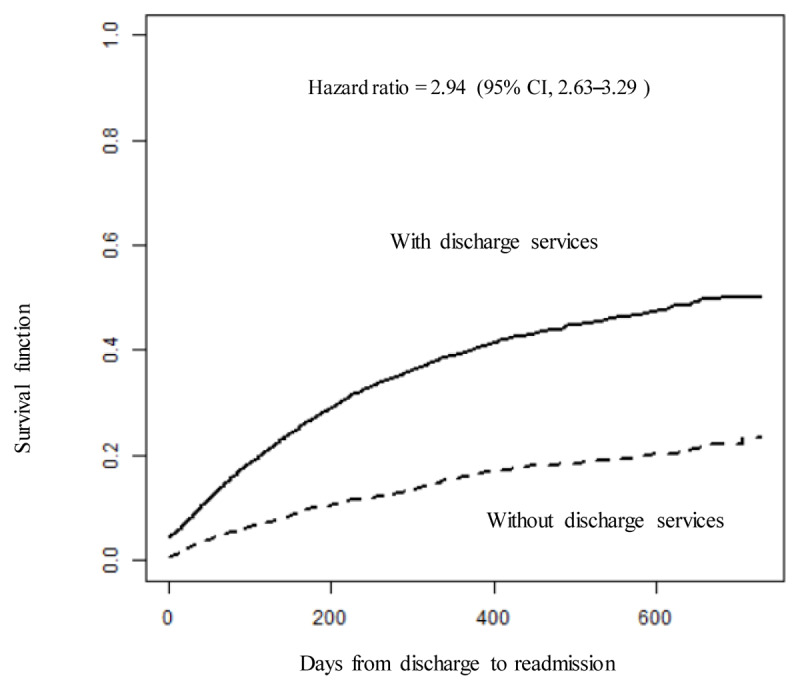
Survival function curve for readmission events with or without discharge services.

Univariable analysis was conducted using the log-rank test, whereas multivariable analysis utilised a Cox proportional hazards model adjusted for age, gender, and the number of complications (≤4, ≥5). For the univariate analysis assessing the days from discharge to readmission with and without discharge services and the corresponding survival function, readmission on day 0 encompassed transfers to acute wards. Detailed results are presented in Table 2 and Figure 2.

**Table 2:** Hazard ratio until readmission event occurs in the group with discharge services and each discharge service compared to the group without discharge services.


	UNIVARIABLE ANALYSIS LOG-RANK TEST				MULTIVARIABLE ANALYSIS COX PROPORTIONAL HAZARDS MODEL			
	
	HR	95% CI		P VALUE	HR	95% CI		P VALUE

Without discharge services	1.00				1.00			

With discharge services	2.94	2.63	3.29	<0.001	2.92	2.6	3.27	<0.001

Age					1.03	1.02	1.03	<0.001

Sex					1.19	1.08	1.3	<0.001

Complications*					1.00	0.91	1.10	0.98

Each discharge service								

Discharge planning	2.15	1.95	2.37	<0.001	2.02	1.83	2.23	<0.001

Age					1.02	1.01	1.03	<0.001

Sex					1.17	1.06	1.28	<0.001

Complications					1.02	0.93	1.12	0.706

Coordination with community care	2.88	2.60	3.19	<0.001	2.83	2.55	3.14	<0.001

Age					1.02	1.02	1.03	<0.001

Sex					1.15	1.05	1.27	0.003

Complications					1.02	0.93	1.12	0.624

Rehabilitation discharge instruction	1.76	1.60	1.95	<0.001	1.77	1.60	1.95	<0.001

Age					1.03	1.02	1.03	<0.001

Sex					1.22	1.11	1.34	<0.001

Complications					1.03	0.94	1.13	0.51


HR = hazard ratio; CI = confidence interval. * Complications are adjusted to 4 or less and 5 or more.

### 3.3 Secondary outcomes: subgroup analysis

Subgroup analysis was conducted based on age, gender, and length of stay categories, calculating HRs for readmission events with the group without discharge services as a reference. Significant differences were observed across all subgroup analyses within the discharge services category: age (65–74-year age category HR = 3.54, 95% CI 2.69–4.67); gender (male category HR = 3.25, 95% CI 2.74–3.86); and length of stay categories (22+ days HR = 3.31, 95% CI 2.50–4.38; <7 days HR = 3.03, 95% CI 2.53–3.64).

Subgroup analysis for each discharge service within similar categories yielded significantly higher readmission HRs. In terms of age, the 65–74-year age category showed the highest HRs: discharge planning HR = 2.86 (95% CI 2.22–3.68), coordination with community care HR = 3.39 (95% CI 2.65–4.35), and rehabilitation discharge instruction HR = 1.82 (95% CI 1.42–2.33). Regarding gender, the male category exhibited higher HRs: discharge planning HR = 2.05 (95% CI 1.76–2.38), coordination with community care HR = 3.08 (95% CI 2.64–3.61), and rehabilitation discharge instruction HR = 1.93 (95% CI 1.66–2.24). For length of stay, the 7–14-day group in discharge planning had a HR = 2.23 (95% CI 1.86–2.67), the <7-day group in coordination with community care had a HR = 3.08 (95% CI 2.64–3.61), and rehabilitation discharge instruction had an HR = 2.24 (95% CI 1.87–2.68). Detailed results are provided in Table 3.

**Table 3:** Subgroup analysis of hazard ratios to readmission event for each category in the group with discharge services and each discharge service compared to without discharge services.


	DISCHARGE SERVICES				EACH DISCHARGE SERVICE											

					DISCHARGE PLANNING				COORDINATION WITH COMMUNITY CARE				REHABILITATION DISCHARGE INSTRUCTION			
			
	HR	95% CI		p-VALUE	HR	95% CI		p-VALUE	HR	95% CI		p-VALUE	HR	95% CI		p-VALUE

Without discharge support	1.00															

Age, years																

65–74 (n = 1,535)	3.54	2.69	4.67	<0.001	2.86	2.22	3.68	<0.001	3.39	2.65	4.35	<0.001	1.82	1.42	2.33	<0.001

75–84 (n = 2,874)	3.07	2.57	3.66	<0.001	2.16	1.85	2.52	<0.001	3.05	2.60	3.58	<0.001	1.74	1.49	2.02	<0.001

85– (n = 2,255)	2.48	2.08	2.96	<0.001	1.68	1.44	1.95	<0.001	2.36	2.01	2.77	<0.001	1.78	1.52	2.08	<0.001

Sex																

Male (n = 2,900)	3.25	2.74	3.86	<0.001	2.05	1.76	2.38	<0.001	3.08	2.64	3.61	<0.001	1.93	1.66	2.24	<0.001

Female (n = 3,781)	2.66	2.29	3.10	<0.001	2	1.74	2.28	<0.001	2.64	2.3	3.03	<0.001	1.67	1.46	1.90	<0.001

Length of stay																

–7 (n = 2,519)	3.03	2.53	3.64	<0.001	2.02	1.67	2.44	<0.001	3.08	2.59	3.66	<0.001	2.24	1.87	2.68	<0.001

7–14 (n = 2,026)	2.84	2.28	3.55	<0.001	2.23	1.86	2.67	<0.001	2.63	2.17	3.19	<0.001	1.64	1.37	1.97	<0.001

15–21 (n = 1,045)	2.28	1.74	2.98	<0.001	1.59	1.27	2	<0.001	2.38	1.87	3.03	<0.001	1.37	1.09	1.72	0.006

22– (n = 1,091)	3.31	2.50	4.38	<0.001	1.99	1.59	2.48	<0.001	3.03	2.36	3.88	<0.001	1.56	1.24	1.97	<0.001


HR = hazard ratio; CI = confidence interval. Hazard ratios were calculated using the Cox proportional hazards model.

### 3.4 Effect of discharge services by confirming the interaction

To ascertain the effect of discharge services by age, gender, and length of hospital stay through subgroup analysis, we examined the interaction of each category (dummy variable) with the discharge service group and each specific discharge service. A significant interaction was observed between the 65–74-year and 85+ age categories in the group receiving discharge services (p = 0.018), indicating a reduction in readmission events in the 85+ age group (HR = 0.68, 95% CI 0.49–0.93). For individual discharge services, a significant interaction was noted in discharge planning and coordination with community care for the 85+ age group compared to the 65–74-year age group (p < 0.1), with fewer readmission events in the 85+ age group: discharge planning (HR = 0.56, 95% CI 0.42–0.75) and coordination with community care (HR = 0.67, 95% CI 0.50–0.91).

A significant interaction was observed in the gender category within the group receiving discharge services (p = 0.090). When comparing the female group to the male group (used as a control), an increase in readmission events was evident (HR = 1.22, 95% CI 0.97–1.53). However, no significant interactions were found within the gender categories for each individual discharge service.

Regarding the length of stay category in the group receiving discharge services, a significant interaction was found between the <7-day and 15–21-day groups (p = 0.058). The 15–21 days group, with the <7-day group as the reference, exhibited a reduction in readmission events (HR = 0.73, 95% CI 0.53–1.01). Significant interactions were noted between all <7-day and 15–21-day groups across each discharge service length of stay category (p < 0.1), indicating fewer readmission events in the 15–21-day group: discharge planning HR = 0.73 (95% CI 0.55–0.98), coordination with community care HR = 0.76 (95% CI 0.56–1.02), and rehabilitation discharge instruction HR = 0.58 (95% CI 0.44–0.78). Further details are presented in Table 4.

**Table 4:** Subgroup analysis of interaction of each category (dummy variable) and the group with discharge services and each discharge service.


	DISCHARGE SERVICES				EACH DISCHARGE SERVICE											

					DISCHARGE PLANNING				COORDINATION WITH COMMUNITY CARE				REHABILITATION DISCHARGE INSTRUCTION			
			
	HR	95% CI		p FOR INTERACTION	HR	95% CI		p FOR INTERACTION	HR	95% CI		p FOR INTERACTION	HR	95% CI		p FOR INTERACTION

Age, years																

65–74 (n = 1,535)	1				1				1				1			

75–84 (n = 2,874)	0.85	0.61	1.18	0.324	0.74	0.55	0.99	0.042	0.88	0.66	1.18	0.402	0.93	0.70	1.24	0.639

85– (n = 2,255)	0.68	0.49	0.93	0.018	0.56	0.42	0.75	<0.001	0.67	0.50	0.91	0.009	0.94	0.70	1.26	0.687

Sex																

Male (n = 2,900)	1				1				1				1			

Female (n = 3,781)	1.22	0.97	1.53	0.090	1.01	0.83	1.23	0.939	1.17	0.95	1.43	0.147	1.16	0.95	1.41	0.151

Length of stay																

–7 (n = 2,519)	1				1				1				1			

7–14 (n = 2,026)	0.92	0.69	1.22	0.559	1.04	0.81	1.34	0.778	0.84	0.65	1.08	0.177	0.72	0.56	0.92	0.009

15–21(n = 1,045)	0.73	0.53	1.01	0.058	0.73	0.55	0.98	0.037	0.76	0.56	1.02	0.066	0.58	0.44	0.78	<0.001

22– (n = 1,091)	1.08	0.77	1.51	0.653	0.92	0.69	1.23	0.593	0.98	0.72	1.32	0.871	0.69	0.51	0.92	0.011


HR = hazard ratio; CI = confidence interval. p < 0.1 for interaction. Hazard ratios were calculated using the Cox proportional hazards model.

## 4 Discussion

The results of this study revealed a higher incidence of readmission events in the group receiving discharge services (HR = 2.92, 95% CI 2.60–3.27). Findings regarding discharge support and readmission in Japan have produced varied results.

### 4.1 Importance of using national health insurance data

Under the Japanese healthcare system, all citizens aged 75 and over are covered by healthcare insurance for the latter-stage elderly, whereas those aged 65 and over who have retired are generally covered by municipal National Health Insurance. Together, these two insurance plans—both of which enrol all citizens not covered by employment-based insurance—form the foundation of Japan’s universal health insurance system. A retrospective, cohort, cross-sectional study investigating the association between discharge support and readmission among all older adults aged 75 and over in Tokyo found no significant results [22]. However, other studies have suggested that discharge support services are linked to lower readmission rates, particularly in community care wards with stringent return-to-home rate criteria [23]. As previous studies were based on data from healthcare insurance for the latter-stage elderly across Tokyo or from a single facility, the observed population may have been either over- or under-represented in assessing discharge support for older adults in a way that reflects the true characteristics of the medical environment. The novelty of the present study lies in its use of survival analysis to examine the association between discharge services and readmission in an acute care ward setting by using National Health Insurance data specific to Kita Ward, Tokyo.

In prior research examining the association with readmission by disease, abnormal laboratory results in elderly patients with cancer [26] and inadequate intervention and treatment for elderly individuals with dementia [27] were identified as significant influencing factors. The patient-specific requirements for diseases encompassed in the present study’s discharge planning were found to be associated with readmissions. The qualitative characteristics of discharge services and the severity of injuries and illnesses were not examined in this study, thus limiting a detailed discussion on the specific factors contributing to readmission. Nonetheless, patient and facility factors related to readmission, encompassed within the requirements of the discharge services, including the institutional context, may have influenced the outcomes. The ensuing discussion will delve into the institutional context associated with readmissions.

### 4.2 Institutional context that influenced readmissions

The breakdown of discharge services revealed that 3,378 individuals (86.5% of the group receiving discharge services) received coordination with community care, underscoring the significant impact of this coordination on the overall readmission rates (HR = 2.83, 95% CI 2.55–3.14). Coordination with community care entails a facility requirement for hospitals to collaborate closely with the community. However, no specific requirements pertain to patients. Elderly patients who received discharge services from healthcare facilities meeting the facility requirements for coordination with community care may have encountered fewer obstacles to readmission. It is posited that an active collaboration with the community within the institutional context facilitates a more receptive approach to readmissions in such facilities.

The discharge planning group comprised patients with challenging discharge factors, such as malignancy, dementia, or acute respiratory infection, and urgent admissions, resulting in an HR of 2.02 (95% CI 1.83–2.23) for readmission occurrence. Prior studies indicated that shorter hospital stays did not correlate with increased readmission rates in patients undergoing surgery for malignancy [28,29]. Additionally, dementia has been proposed as a potential factor for readmission due to progression of the underlying condition [30,31]. High-risk factors for readmission for the group receiving discharge services may extend beyond specific diseases. Thus, it is crucial to consider not only patient requirements but also facility-related requirements.

Higher readmission rates have been observed for discharges to home with home care compared to discharges to nursing homes [32]. In addition to medical institutions offering discharge services, home healthcare services that monitor patients’ progress following hospital discharge should be recognised as a significant facility-related risk factor for readmission. Conversely, a significantly greater number of patients in the group receiving discharge services died at home under the care of a home physician (p < 0.001). This observation suggests that discharge services may be intricately linked to end-of-life care.

### 4.3 Effects of discharge services

The purpose of discharge services for elderly patients proposed by the Japanese government is to enable them to continue living in their familiar communities. Although the prevention of hospital readmission is internationally recognised as a standard medical objective, discharge services in Japan do not necessarily aim to prevent readmissions.

This study used a combination of multivariate and subgroup analyses by applying the Cox proportional hazards model to account for confounding biases between discharge services and readmission. Subgroup analyses based on age, gender, and length of hospital stay revealed an interaction effect between the 65–74-year age group (HR = 1) and the 85+ age group (p for interaction = 0.018, HR = 0.68, 95% CI 0.49–0.93), indicating that discharge services might effectively reduce hospital readmissions for the 85+ age group. The reduction in readmission HRs observed in the 85+ age group compared to the 65–74-year age group aligns with statistical trends [33], indicating that age-specific hospitalisation rates (number of admissions per 100,000) in the older age group exhibit a declining trend annually.

This study could not provide an explanation for the observed effect of discharge services on readmission prevention specifically for the age group of 85 years and older, nor for those with a hospital stay of 15–21 days. However, regarding age, previous research has suggested a tendency for healthcare costs to shift towards long-term care expenses beyond a certain age. This implies that aggressive treatment in acute care wards may have been limited, with care potentially replaced by home nursing and similar services [34]. On the basis of these findings, we propose enhancing remuneration for long-term care expenses, including home-visiting nursing services, following discharge from acute care wards for individuals aged 85 and older. Establishing a supportive environment for home-based care is expected to facilitate smoother adaptation to daily life immediately after discharge.

Regarding gender, using the group without discharge services as a reference (HR = 1), males experienced a significantly higher incidence of readmission events (HR = 3.25, 95% CI 2.74–3.86). However, subgroup analyses revealed no significant interaction between each discharge service intervention and gender (p for interaction = 0.147–0.939). Men in the discharge services group may have had some factors associated with readmission.

The results of subgroup analysis concerning the length of stay indicated a significant interaction solely in the 15–21-day hospital stay group (p for interaction = 0.058, HR = 0.73, 95% CI 0.53–1.01) compared to the <7-day hospital stay group, suggesting potential factors linked to hospital readmission in the discharge service group. The discharge planning intervention stipulated planning within 3 or 7 days of admission as part of the calculation requirement. However, a shorter length of stay emerged as a high-risk factor for readmission, suggesting that the duration of appropriate discharge services may reduce readmissions.

The Japanese government advocates for a reduction in hospital stays. However, to provide sufficient decision-making support to patients and their families and to coordinate effectively with local healthcare and long-term care facilities, a certain period of time is necessary. For those responsible for managing bed occupancy in acute care wards, who may feel burdened by demands for shortened hospital stays, we hope the findings of this study will serve as a foundation for more effective support.

The results of this study reflect the situation in Kita Ward, Tokyo, where there is an abundance of healthcare resources, suggesting that discharge services have facilitated collaboration among healthcare institutions, thereby reducing barriers to hospital readmission.

### 4.4 Study limitations

Based on the reality of discharge services in a multicentre setting using local authority reimbursement data, this study showed an association with readmission after discharge of elderly patients. The strength of this study is its longitudinal analysis of the effects of discharge services. However, there are a number of limitations in the reimbursement data, which are presented below to describe the limitations of this study.

Characteristics of the specific municipal reimbursement data used in this study are that they do not include cases transferred to hospital or scheduled or planned admissions, and they do not confirm the name and severity of the main injury or illness. In addition, it is not possible to distinguish cases in which a hospitalisation deemed to be a first admission was actually a readmission in the data extraction period limited to two years. Furthermore, the quality of discharge services at each medical institution is not taken into account and may be difficult to apply in other regions with different social resource environments, including healthcare- and welfare-related facilities. In the target population, the patient’s wishes, the family care system, and the residential and geographical environment also have not been considered. Due to the nature of the data, there are limitations in controlling for validation steps and confounding variables.

In the community-based integrated care system, which is regarded as the key to integration in medical care in Japan, the focus is on discharge services for elderly people who have completed treatment and require nursing care, for which they are transferred from an acute care ward to a convalescent/recuperative hospital or a community-based integrated care ward, and then returned to their familiar residential environment. This study focused on elderly patients who received discharge services in acute wards, but further studies are needed by function of the medical institution. In addition, further analysis that includes the characteristics of the diseases and care systems related to readmission should be carried out to develop studies on the establishment of a community-based integrated care system according to the characteristics of the community.

## 5 Conclusion

The results of this study suggest that discharge services in a community-based integrated care system may increase the demand for readmissions. However, they also suggest that discharge services may reduce readmissions for people aged 85 years or older and those with a medium average length of hospital stay.
